# Progress in Diseases of the Blood

**Published:** 1903-07-04

**Authors:** 


### Progress in Diseases of the Blood.

(*Concluded from page* 231.)

**Leukæmia.**—Dorothy Reed[4] reports a case of acute lymphatic leukæmia without enlargement of the lymph glands. From an exhaustive microscopic examination of the bone-marrow and lymphadenoid tissues she concludes that the prevalent cells found in the blood were derived, not from the lymph glands, as has been hitherto supposed, but from the bone-marrow, which was in a condition of hyperplasia and gave evidence that the so-called lymphocytes originated here. The lymphatic glands, though not appreciably enlarged, showed "infiltration" by lymphoid cells, which she was able to distinguish by their morphological characters, and staining properties from the proper cells of the glands, and she concludes that the enlargement occurs when this (secondary) infiltration has gone sufficiently far. No evidence was forthcoming that the glands were excessively active, and the writer holds that there is little evidence extant to establish the view that the true lymphocyte arises in the lymph gland. She finds difficulty in believing that the non-amœboid lymph cells can leave the lymph glands and enter the blood-stream *viâ* the thoracic duct, as the anatomical structure is against this. The question of amœboid movement in the lymphoid cells is discussed; a case of lymphatic leukæmia is quoted in which these cells were shown to possess the power of movement, though Ehrlich has denied its existence in them. Perhaps this explains the infiltration of the lymph glands in this disease, as it is commonly seen; while in the spleno-medullary form the non-amœboid myelocyte does not leave the blood-stream to wander into the lymphatic system, though it can infiltrate the liver. In the bone-marrow, "the similarity in form of the small lymphoid cells with the normoblasts, which were conspicuously few in number, was suggestive. It is not impossible that the increase here was in another cell, from which the lymphoid cell and the hæmoglobin-containing cell are normally derived." The writer finds evidence in support of this suggestion in cases of other forms of anæmia which she has examined. Adducing cases recorded by other observers, she contends that the lymphatic and myelogenous (spleno-medullary) forms are both due to proliferation of marrow cells, that both forms may be acute or chronic, and that mixed-celled forms are seen more frequently, indeed, than the pure form of either. As a more scientific nomenclature she suggests that the varieties might be called myelocytic, lymphoid, and mixed-celled, according as one or the other type of cell predominates in the blood-picture. Warthin[5] draws attention to the condition of the prævertebral hæmolymph glands and spleen in pernicious anæmia. From an examination of eight cases he concludes that these structures play an important part in hæmolysis, though their state is not peculiar to pernicious anæmia, as it may be produced by other infections or toxic processes which cause great hæmolysis. A case is reported by A. F. Edwards[6] which showed the clinical signs of aneurysm of the descending aorta either just above or just below the diaphragm, with marked expansile pulsation of the chest-wall and symptoms of disturbance of the liver. The blood, too, showed severe anæmia, and the hæmoglobin index was $\frac{32}{24}$. No evidence, however, was found *post-mortem* to account for the clinical symptoms, and especially the pulsation. The vessels were atheromatous, but there was no aneurysm or dilatation, but some adhesions of the pleura, and the diagnosis arrived at from the state of the blood, bone, and marrow was that of pernicious anæmia. G. Finzi[7] thinks that a factor in the production of pernicious anæmia may be any extensive change in the mesenteric glands, which of course assist in the formation of the chyle. These changes may be due to various infections of the intestinal mucosa. Max Einhorn[8] discusses the old theory of Flint that pernicious anæmia may be due to achylia gastrica or atrophy of the stomach, and decides against it, because (1) in most cases of achylia the blood is nearly normal, and (2) gastric juice is found at any rate in some cases of the former disease. In a few instances, no doubt, both diseases are present together from common causes, or because the anæmia leads to changes in the stomach just as it does in the cord. He brings forward three instances where a state of achylia returned in time to one of normal secretion, showing that the disease is not necessarily of lifelong duration, as has been supposed. Bret and Cade[9] deny that the liver has any part in the genesis of pernicious anæmia, and found identical changes in that organ in each of three cases which they examined. Thus there were necrosis of cells and exudation from the capillaries at the centre of a lobule with commencing fibrosis, little or no fatty change, but always a deposition of granules of iron at the periphery. They also noticed an infiltration of leucocytes in the portal spaces and larger trabeculæ, but this was not universal.

**Lead Poisoning.**—Boston[10] in 24 cases found a leucocytosis existing in all but three, and of these three two were of long standing and one had been for some time under treatment, which suggests that the increase is only produced in the primary stage. Cabot's 15 cases averaged 12,900 white cells, and these gave 12,600. The differential counts showed normal proportions. The red cells were slightly diminished with poikilocytosis, many macro- and micro-cytes, and some nucleated red cells. From the results of another case of plumbism Wolff[10] argues that the spleen can take on vicarious myeloid functions in place of the bone-marrow. There appeared to be insufficient action of the latter, while the blood showed many neutrophile leucocytosis; but the spleen contained numerous granular cells which were in fact myelocytes, as well as nucleated red cells.

**Splenic Anæmia.**—A special variety is described by Weil and Clerc.[10] Besides the anæmia and the enlargement of the spleen there is a peculiar blood state, showing myelocytes from 1·5 to 29 per cent. and numerous nucleated red cells without any leucocytosis. There is little or no enlargement of the liver and other glands. The hæmoglobin is greatly reduced, and the red cells may fall below 1,000,000. The disease commences insidiously, but runs a fatal course in a few months. It presents points of resemblance to both pernicious anæmia and myelogenous leucocythæmia, though it is distinct from either. C. W. Field[11] reports a case of Banti's disease in which, besides the splenic, liver, and blood changes, there was typical interstitial nephritis. Kidney disease is mentioned cursorily by Banti in some of his cases, but he does not appear to connect it with the other lesions.

**Leucocytosis in Appendicitis.**—G. L. Gulland[12] gives an interesting analysis of recent views on the value of the blood-count as a guide to operation. His conclusions are, that if a count of 20,000 or more is found operation is demanded, unless the count rapidly falls with improvement of the symptoms. If the count is below 20,000, a differential count of 500 cells should be made, and then, if 80 per cent. are polymorphonuclear, operation is called for ; if they are below 70, it will probably not be needed. If the iodine reaction of Ehrlich be marked, operation is probably always indicated, except in hopeless cases of general sepsis with a low leucocyte count.

[4] Amer. Jour. Med. Sci., Oct. [5] Ibid. [6] Ibid. [7] Med. Rec., Feb. 7. [8] Med. Rec., Feb. 28. [9] Brit. Med. Jour., Dec. 20. [10] Brit. Med. Jour., Feb. 28. [11] Brit. Med. Jour., Feb. 7. [12] La Semaine Med. and Med. Chron., Jan. [13] Amer. Jour. Med. Sci., March. [14] Scot. Med. and Surg. Jour., Feb.

